# Greffe de cornée: expérience du service d'ophtalmologie au CHU Mohammed VI de Marrakech, Maroc

**DOI:** 10.11604/pamj.2016.23.158.8667

**Published:** 2016-04-06

**Authors:** Sarah Belghmaidi, Ibtissam Hajji, Hasna Soummane, Wiam Ennassiri, Hafsa Essafi, Abdeljalil Moutaouakil

**Affiliations:** 1 Service d'Ophtalmologie, CHU Mohammed VI, Marrakech, Maroc

**Keywords:** Kératoplastie, cornée, indications, rejet, kératocône, Keratoplasty, cornea, indications, rejection, keratoconus

## Abstract

La kératoplastie transfixiante est l'une des plus fréquentes des greffes de tissus et transplantations d'organes. Le but de notre travail est de rapporter notre expérience en matière de greffe de cornée. C'est une étude prospective de 195 patients ayant bénéficié d'une greffe de cornée sur une période allant d'aout 2009 à Août 2015. Le recul moyen est de 21 mois. Les indications de la greffe étaient le kératocône 33.8%, les séquelles de traumatisme 10.7%, la kératopathie bulleuse 19.5%, les dystrophies héréditaires 10.7%, et les kératites herpétiques 9.2%. L'acuité visuelle initiale était inférieure à 1/10 dans 90% des cas. Nous avons observé une réaction de rejet dans 19 yeux dont 14 étaient récupérables, 33 hypertonies oculaires, 20cataracte, et 2 décollements descmetiques. La greffe de cornée apparaît comme une intervention donnant de bons résultats anatomiques et fonctionnels. Les résultats ne doivent pas occulter une surveillance post opératoire étroite et régulière pour dépister à temps d’éventuelles complications, en particulier le rejet de greffe.

## Abstract (en)

Penetrating keratoplasty is one of the most common tissue grafts and organ transplantations. The aim of our study was to report our experience in corneal transplantation. A prospective study of 195 patients who underwent corneal transplantation over the period from August 2009 to August 2015. Mean follow-up was 21 months. The indications for transplantation were keratoconus 33,8%, trauma-related sequelae 10,7%, bullous keratopathy 19,5%, hereditary dystrophies 10,7% and herpes keratitis 9,2%. Initial visual acuity was less than 1/10 in 90% of the cases. We observed corneal graft rejection in 19 eyes (14 were recoverables), 33 ocular hypertonia, 20 cataracts, 2 without significant pathological history. Corneal transplantation appears as a surgical intervention giving good anatomical and functional results. The results should not obscure a close and regular post operative monitoring to screen for possible complications, especially graft rejection.

## Introduction

La kératoplastie transfixiante consiste à remplacer un disque cornéen central de pleine épaisseur. C'est l'une des plus fréquentes des greffes de tissus et transplantations d'organes [1]. La greffe de cornée est une intervention relativement simple, peu coûteuse comparée aux transplantations d'organes. Si elle répond aux mêmes lois générales de la transplantation que les autres allogreffes, elle bénéficie d'un privilège immunitaire. Les transplantations au Maroc restent insuffisantes. Pour remédier à cette situation, le Maroc s'est engagée dans un programme de promotion de greffes d'organes et de tissus avec un objectif de greffes de cornée de 1.000 par an. «La création d'une banque de tissus, l'engagement des procédures pour la diversification de l'importation des greffons de cornées, l’élaboration des règles de bonne pratique, la mise à jour des contre-indications de la greffe de la cornée, la promotion du don d'organes et de tissus, le prélèvement d'organes sur donneur décédé ainsi que l'opérationnalisation des lois et textes régissant la greffe de la cornée.» L'objectif de notre étude est de: rapporter notre expérience en matière de greffe de cornée au sein du CHU Mohammed VI de Marrakech, Maroc.

## Méthodes

Il s'agit d'une étude prospective portant sur 195 cas ayant bénéficié d'une greffe de cornée au sein de service d'ophtalmologie du CHU Mohamed VI de Marrakech sur une période allant d'Août 2009 jusqu'en Août 2015. Parmi ces 195 greffes, 40 greffons étaient prélevés localement, les autres ont été importés. Pour mener cette étude, une fiche d'exploitation a été établie, remplie par l'investigateur où il a été noté: L'identité, les antécédents, les signes cliniques notamment l'acuité visuelle et l'examen de la cornée, et para cliniques, la technique opératoires les complications et le suivi des patients à court et à long terme. Les patients à haut risque de rejet ont bénéficié d'une préparation préopératoire rigoureuse: traitement des néovaisseaux cornéens, prévention anti herpétique, bolus de méthylprédnisolone chez les candidats ayant plus d'un facteur de risque. Les différentes interventions réalisées au sein de notre formation sont: la kératoplastie transfixiante, la kératoplastie lamellaire antérieure profonde et la greffe-bouchon. Le geste était parfois associé à d'autres procédures selon les indications notamment une extraction extracapsulaire, implantation, ou une vitrectomie. Tous nos patients ont reçu une corticothérapie topique, des agents mouillants, et une antibiothérapie topique en post opératoire. Le suivi post opératoire se fait chaque jour jusqu'au J7, chaque semaine jusqu'au M3, chaque mois jusqu'au 1 an et puis chaque 3 mois jusqu'au 3 ans. Les calculs statistiques ont été réalisés à l'aide des logiciels SPSS 10.0 pour le Windows.

## Résultats

**Figure 1:**
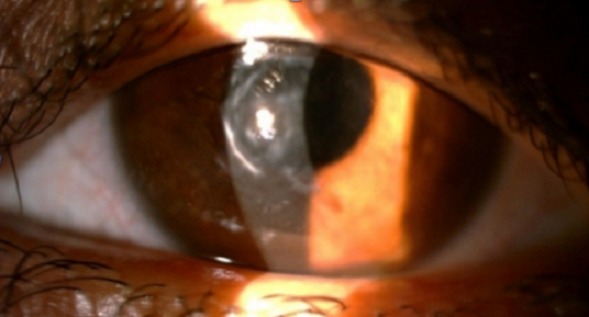
Patient présentant un kératocône évolué avec opacité

**Figure 2:**
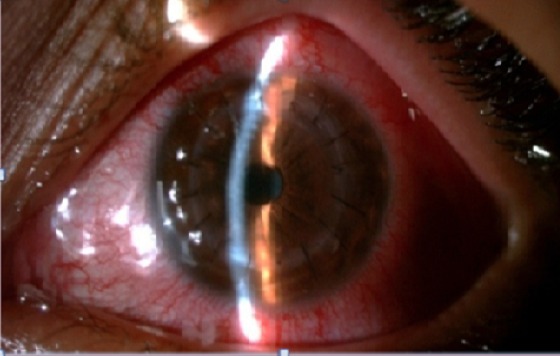
Patient bénéficiant d'une kératoplastie

**Figure 3:**
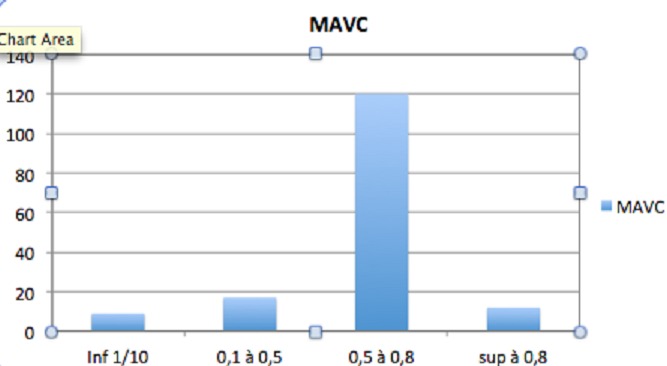
Évolution de l'acuité visuelle après greffe de cornée

Le recul moyen est de 21 mois. Dans notre série, 195 yeux de 90 femmes et 95 hommes ont été étudiés. L’âge moyen au moment de l'intervention était 34 ans allant de 6 à 85 ans. 66 yeux greffés présentaient un kératocône (33,8%) (Figure 1); 21 taies post-traumatiques (10,7%); 36 Kératopathies bulleuses (19,5%); 21 dystrophies héréditaires (10,7%); 18 séquelles kératites herpétiques (9,2%); 12 taies post infectieuses (6,4%) et 21 autres indications (10,7%). 38 candidats ont reçu un traitement pour néovascularisation cornéenne en pré opératoire: 17 ont été mis sous corticothérapie, 13 ont bénéficié d'une injection sous conjonctivale du bévacizumab et 18 d'une injection intra stromale du même produit. Le but de la kératoplastie était optique chez 177 patients et architectonique chez 17. Il s'agissait d'une intervention simple chez 151 patients (77,4%), combinée à une triple procédure (extraction+ implantation+ greffe) chez 30 (15,3%), et séquentielle chez 14 (7,2%). Nous avons réalisé 185 kératoplasties perforantes (Figure 2), 3 kératoplasties lamellaires antérieures profondes, et 7 greffes bouchon. Nous utilisons des points séparés pour suturer nos greffons. Le diamètre de trépanation moyen était 7,50 mm. La meilleure acuité visuelle corrigée avant la greffe (MAVC) était inférieure à 1/10 chez 90% des patients; et inférieure à 2/10 chez 100% (moyenne LogMar 1,2). La MAVC finale moyenne post opératoire était 0.3, 60% avaient une MAVC supérieure à 5/10 (Figure 3). En étudiant les différents facteurs astigmatogènes: l’étiologie la plus astigmatogène est le traumatisme. L'excentricité du greffon constitue le principal facteur peropératoire engendrant un astigmatisme géant. En post opératoire, le décalage des berges reste un facteur d'astigmatisme mal contrôlable. L'ablation sélective des fils topoguidée a été démarrée après 6 mois et a permis une dégression des valeurs moyennes de l'astigmatisme (de 6 à 4,50 D), La diminution a été plus significative avant un an. L'ablation totale de sutures a été réalisée à 13 mois du postopératoire en moyenne. L'astigmatisme moyen final était 4,25 D. Concernant les moyens de correction optiques: 160 ont été corrigés par lunettes, 22 par lentilles rigides et 6 n'ont pas nécessité de correction. 15% des patients ont été repris. Le délai d’épithélialisation moyen était 4 jours allant d'un à 18 jours. Sur la période de suivi, 19 rejets ont été constatés,Le traitement associait une hospitalisation, une corticothérapie locale intensive et générale par bolus intraveineux. 14 cas ont été traités et 5 étaient irrécupérables. La pression intraoculaire (PIO) préopératoire mesurée par aplanation était inférieure à 18 mmHg chez tous les patients. 33 cas (16,9%) ont présenté une HIO en postopératoire qui a pu être contrôlée par un traitement local unique ou en association. Aucun cas d'endophtalmie, ni de kératite n'a pas été rapporté. 20 cas de blépharites ont été observés et ont été traités médicalement par des soins de paupières et un antibiotique local. 20 patients (10,2%) ont développé une cataracte. 2 cas de décollements descemétiques ont été observés. Aucune complication du segment postérieur n'a été observée a part un décollement de rétine.

## Discussion

L’âge moyen de nos patients était de 34 ans avec des extrêmes allant de 6 à 85 ans. Selon Donald T, les patients d’âge jeune (21 - 40 ans) avaient un meilleur pronostic par rapport aux patients âgés vu la faible réserve en cellules endothéliales et par rapport aux enfants vu le risque accru de rejet [2]. La prédominance masculine a été notée par plusieurs les auteurs, selon Al-yousuf N il y a une relation significative entre le sexe masculin et le kératocône [3]. La fréquence de différentes indications dans notre étude montre la prédominance du kératocône suivi de la kératopathie bulleuse. Rahmans [4] et Legeais [5], retrouvent également le kératocône comme première indication de kératoplastie transfixiante dans leurs séries respectives, alors que les données de la littérature [2, 6, 7] montrent une progression dans la fréquence des kératoplasties pour Kératopathies bulleuses à partir des années 80, correspondant aux complications des implants intraoculaires. Les traumatismes perforants représentent une indication majeure pour la KT, en raison de leurs séquelles et la fréquence des accidents dans notre contexte. Selon une étude de Wiliams portant sur un large échantillon de 1866 kératoplasties transfixiantes avec un suivi allant d'un an à 22 ans 24: la survie du greffon est respectivement à un, cinq, dix et quinze ans de 87%, 73%, 60% et 46%. Selon les indications: le taux de survie est de 98% à 5 ans pour le kératocône, 86% pour les dystrophies cornéennes, 75% pur les kératites herpétiques, et 70% pour les traumatismes cornéens [8]. La vitalité du greffon est également liée à la qualité du limbe scléro-cornéen et au nombre de cellules souches limbiques. Compte tenu des risques de la greffe de cornée, il est communément admis que la greffe n'est proposée que devant une acuité visuelle corrigée inférieure à 2/10. Dans notre expérience la majorité des patients avaient une acuité visuelle à 1 /10 et ceci s'explique par le taux élevé de kératocône stade avancé. L'acuité visuelle postopératoire est fonction de la qualité du greffon, mais également de la transparence du cristallin ou de la présence d'un implant, de l’état de macula et du nerf optique. La récupération visuelle post-greffe est très progressive, la vision est faible le premier mois postopératoire, liée au temps de déturgescence du greffon. L'amélioration de l'acuité visuelle s’étale sur deux ans voir plus.

Dans notre étude, 60% des cas avaient une MAVC supérieure à 5/10. Selon la littérature [9], le kératocône avec la dystrophie de Fuchs s'accompagnaient de bons résultats fonctionnels contrairement à la kératopathie bulleuse, les séquelles infectieuses et les taies post traumatiques. Le gain visuel dépend de la sphère et de l'astigmatisme postopératoire. L'importance de l'astigmatisme dépend de la gestion des sutures en per-opératoire et en postopératoire. Un mauvais affrontement des berges provoque une modification des courbures et des diamètres cornéens. Aucune complication peropératoire n'a été notée dans notre série. Le délai moyen de réépithélialisation était de 4 jours allant d'un jour à 18 jours et tout retard était en rapport avec l’âge du donneur et la présence d'un diabète. Le rejet d'allogreffe de cornée, est la première cause d’échec des kératoplasties transfixiantes [1].17 cas de rejets étaient observés dans notre série, nos résultats sont comparables avec les données de la littérature [10–12]. Le traitement curatif du rejet doit être le plus précoce possible afin de minimiser la perte cellulaire endothéliale induite par le rejet et ainsi augmenter les chances de récupération de la transparence du greffon après traitement. L'hypertonie oculaire concerne dans notre expérience 16,9% de sujets opérés de kératoplastie. Selon les données de la littérature, la fréquence des hypertonies après greffe de cornée est très variable selon que l'on considère, l'atteinte papillaire, l'hypertonie chronique ou toute hypertonie supérieure à 21 mmHg [13]. Les complications infectieuses ont été limités dans notre étude: 20 cas de blépharites, aucun cas d'endophtalmie ni de kératite virale. La fréquence observée des complications microbiennes varie de 1,8% à 11,9% aprèsgreffe de cornée toutes étiologies confondues [14]. La survenue de la cataracte est imputée à la durée de la corticothérapie locale. 10,2% de nos patients ont présenté une cataracte, Lim et al [15] ont rapporté un taux de 4,5% après le suivi de 93 yeux sur 4 ans.

## Conclusion

La kératoplastie transfixiante apparaît comme une intervention donnant de bons résultats anatomiques et fonctionnels. Les résultats ne doivent pas occulter une surveillance post opératoire étroite et régulière pour dépister à temps d’éventuelles complications, en particulier le rejet de greffe. La sélection des indications et le traitement préventif du rejet permettraient d'améliorer les résultats de la greffe de cornée.

### Etat des connaissance sur le sujet

- La greffe de cornée est une technique connue.

### Contribution de notre étude a la connaissance

- C'est la première expérience au Maroc spécialement à Marrakech. Première greffe faite en 2008. Difficultés dans les pays en voie de développement.
